# Genetic alterations and allele frequency of* BRAF V600E* and *TERT* mutation in papillary thyroid carcinoma with intermediate-to-high recurrence risk: a retrospective study

**DOI:** 10.1007/s10238-024-01320-4

**Published:** 2024-04-12

**Authors:** Jiayu Huang, Jiazhi Wang, Jingchao Xv, Jingran Wang, Guangzhi Wang, Yongfu Zhao

**Affiliations:** Department of Thyroid Surgery, The Second Hospital of Dalian Medical University, Dalian, Liaoning China

**Keywords:** Papillary thyroid carcinoma, *BRAF V600E* mutation, *TERT* mutation, Allele frequency, Recurrence

## Abstract

The predictive value of allele frequency (*AF*) of *BRAF V600E* and *TERT* mutations in papillary thyroid carcinoma (PTC) remains controversial. We aimed to investigate the *AF* of *BRAF V600E* and *TERT* mutations in intermediate-to-high risk PTC and their association between tumor invasiveness, prognosis, and other mutations. Probe hybridization capture and high-throughput sequencing were used to quantitatively test 40 gene loci in 94 intermediate-to-high recurrence risk PTC patients, combined with clinical characteristics and follow-up for retrospective analysis. *BRAF V600E* mutation *AF* was linked to a increased risk of thyroid capsule penetration, recurrence, and concurrent mutations. Concurrent mutations could lead to a worse prognosis and increased invasiveness. *TERT* promoter mutation frequently accompanied other mutations and resulted in a poorer prognosis. However, there was no clear association between the *TERT* mutation *AF* and tumor invasiveness or recurrence. The sensitivity and specificity of predicting recurrence in intermediate-to-high risk PTC with *BRAF V600E* mutation *AF* > 28.2% were 60 and 80%. Although genetic alterations in PTC can differ among different ethnicities, the *AF* of *BRAF V600E* and *TERT* mutations may be similar. The *AF* of *BRAF V600E* has the potential to be a novel indicator in predicting PTC invasiveness and prognosis.

## Introduction

In recent decades, there has been a global increase in the occurrence of thyroid cancer [1]. Papillary thyroid carcinoma (PTC) is the most frequent type of thyroid cancer, and the *BRAF V600E* mutation is the most frequent genetic mutation in PTC [2]. The BRAF gene encodes B-Raf protein kinase, which is a crucial member of the MAPK signaling pathway that regulates cell proliferation and differentiation [3]. Research suggests an association between PTC with *BRAF V600E* mutation and tumor volume, invasiveness and metastasis [4, 5]. However, it remains controversial whether *BRAF V600E* accurately reflects the malignancy and prognosis of the tumor [6–8]. The *TERT* gene encodes telomerase, which is a crucial enzyme for maintaining chromosomal telomere length. Activation of *TERT* gene mutations can sustain telomere length, leading to unlimited cell proliferation. Research has shown that mutations in the *TERT* gene are related to increased tumor size, invasiveness, lymph node metastasis and resistance to radiotherapy [9, 10]. However, the severity of PTC with *BRAF V600E* mutation can be variable, and patients with BRAF mutations may also present with different conditions, possibly related to the individual frequency of *BRAF V600E* in each patient [9]. Allele frequency (*AF*) is calculated by dividing the number of mutated molecules by the total number of wild-type molecules at a specific position in the genome [11, 12]. In this retrospective review of gene test results from Chinese patients with intermediate-to-high recurrence risk PTC [13], we analyzed and explored the impact of *BRAF V600E* and *TERT* mutation *AF*, aiming to identify reliable indicators that reflect the invasiveness and prognosis of PTC.

## Materials and methods

### Patients

A retrospective analysis was conducted on 106 patients with thyroid cancer who underwent genetic sequencing at the Department of Thyroid Surgery, The Second Hospital of Dalian Medical University, from November 2018 to December 2023. The inclusion criteria were: (1) postoperative pathology confirming PTC and (2) intermediate or high-risk stratification for PTC recurrence, stratified according to the ATA initial recurrence risk stratification [13]. 94 patients who met the inclusion criteria were included. The data collected included age, sex, stage, pathology, genetic testing results, and follow-up outcomes.

### Second-generation sequencing

The gene testing was conducted by Beijing Genetron Health Co., Ltd. The Genetic testing loci was shown in Table 1. The steps of the testing process are as follows:

1. DNA Extraction: DNA concentration was measured using Qubit dsDNA HS Assay Kits (Thermo Fisher Scientific, Q32851), and RNA concentration was measured using Qubit RNA HS Assay Kits (Thermo Fisher Scientific, Q32852).
2. Reverse Transcription and PCR Amplification: To prepare the reaction system, take 0.2-mL PCR tubes equal to the number of TNA samples + 2. Total TNA 100 ng, make up to 7 µL with nuclease-free water. For RNA heat treatment, use the following reaction conditions: 80 °C for 10 min followed by 25 °C for 3 min. Then add 1μL of reverse transcriptase solution and 2μL of reverse transcriptase buffer solution to the reaction, mix well, and place on ice. Finally, set the PCR machine program with synthesis conditions. Perform PCR amplification by taking out the cDNA amplification primer mix, specific adapter, and polymerase mix, melting, mixing and following the temperature and time conditions of 25 °C for 10 min, 42 °C for 60 min, and 85 °C for 5 min.
3. Library Construction: To mix the magnetic beads, place them at room temperature for 30 min, vortex to disperse the beads, and slowly aspirate the solution. Next, prepare an 80% ethanol solution. Add 3μL of DNA library from pool 1 to pool 2 in a 3:20 ratio, shake well, let it stand at room temperature for 5 min, centrifuge briefly, place on a magnetic stand for about 5 min until the liquid clears, and discard the supernatant.
4. Library Quantification: The library concentration should be quantified using the Qubit fluorescence quantification system with the recommended DNA nucleic acid quantification reagent kit. Additionally, the library concentration and band size should be quantified using Agilent 2100/2200/4200. A target fragment size of around 180-300 bp is recommended, and the dimer proportion should not exceed 25% to be considered qualified. Sequencing should be performed on samples using the GENETRON S5 gene sequencer produced by Genergy Bio Technology. The BaseCaller software is utilized for base identification and base information statistics, and the TMAP software is used to align the sequencing results to the human reference genome sequence hg19 (GRCh37). Bam files and data statistics are generated for all samples on the chip.
5. Quality Control Conditions: For DNA samples: Average sequencing depth (Mean Depth) ≥ 5000x; *BRAF V600* sequencing depth ≥ 2000x; *TERT* amplicon average sequencing depth ≥ 1000x. For RNA samples: The number of reads aligned to the target region ≥ 20,000; At least 3 out of 5 reference genes have a read count ≥ 50.

**Table 1:** Genetic testing loci (*n* = *40*)

DNA genetic mutation testing loci			
*AKT1*	*EZH1*	*KRAS*	*SPOP*
*BRAF*	*GNAS*	*NRAS*	*TERT*
*CTNNB1*	*HRAS*	*PIK3CA*	*TP53*
*EIF1AX*	*TSHR*	*RET*	*ZNF148*
RNA gene fusion testing loci			
*AFAPIL2-RET*	*EML4-NTRK3*	*KIAA1549-BRAF*	*SQSTM1-RET*
*CCDC186-RET*	*IRF2BP2-NTRK1*	*OSBPL9-BRAF*	*THADA-LOC389473*
*EML4-ALK*	*BRAF-OSBPL9*	*SQSTM1-NTRK3*	*NTRK1-TPM3*
*GFPT1-ALK*	*CREB3L2-PPARG*	*THADA-IGF2BP3*	*PRKAR1A-RET*
*AKAP9-BRAF*	*ETV6-NTRK3*	*NCOA4-RET*	*STRN-ALK*
*CCDC6-RET*	*KIAA1217-RET*	*PAX8-PPARG*	*TPM3-NTRK1*

### Statistical analysis

The analysis was performed using Statistical Package for Social Sciences (SPSS) version 29.0. Descriptive and frequency analyses were conducted, and the distribution was represented using frequencies, means, and standard deviations. Nonparametric tests or chi-square tests were employed for data that did not meet the assumption of normal distribution or had a small sample size, making normality tests impractical. Conversely, data conforming to normal distribution underwent analysis of variance or t tests. Logistic regression analyses were used to analyze *AF* between clinical statistics. The ROC curve was used to determine the optimal critical value. The confidence interval was based on 95%, and the level of statistical significance was *p* value < 0.05.

## Results

This study included 94 patients with intermediate-to-high recurrence risk in PTC, with a male-to-female ratio of 1:1.35. The mean age was 43.99 ± 15.35 years, and the average follow-up time was 1.90 ± 1.75 years. No distant metastases were detected in patients during diagnosis or follow-up. Among these patients, 88 (93.62%) patients exhibited at least one genetic alteration. 78 (82.98%) patients had gene mutations, 11 (11.70%) patients had gene fusions, 1 (1.06%) patient had both gene mutation and fusion, and 6 (6.38%) patients showed no genetic abnormalities. The gene mutation events observed included *BRAF V600E* missense mutation (*n* = 75), *TERT* promoter mutation (*n* = 17), AKT1 missense mutation (*n* = 1), KRAS missense mutation (*n* = 1), TP53 missense mutation (*n* = 1), PIK3CA missense mutation (*n* = 2), and HRAS missense mutation (*n* = 1). The gene fusion events observed in this study included CCDC6-RET gene fusion (*n* = 7), NCOA4-RET gene fusion (*n* = 2), and ETV6-NTRK3 gene fusion (*n* = 2).

### Association between* AF *of *BRAF V600E* mutation and gender/age

**Fig. 1:**
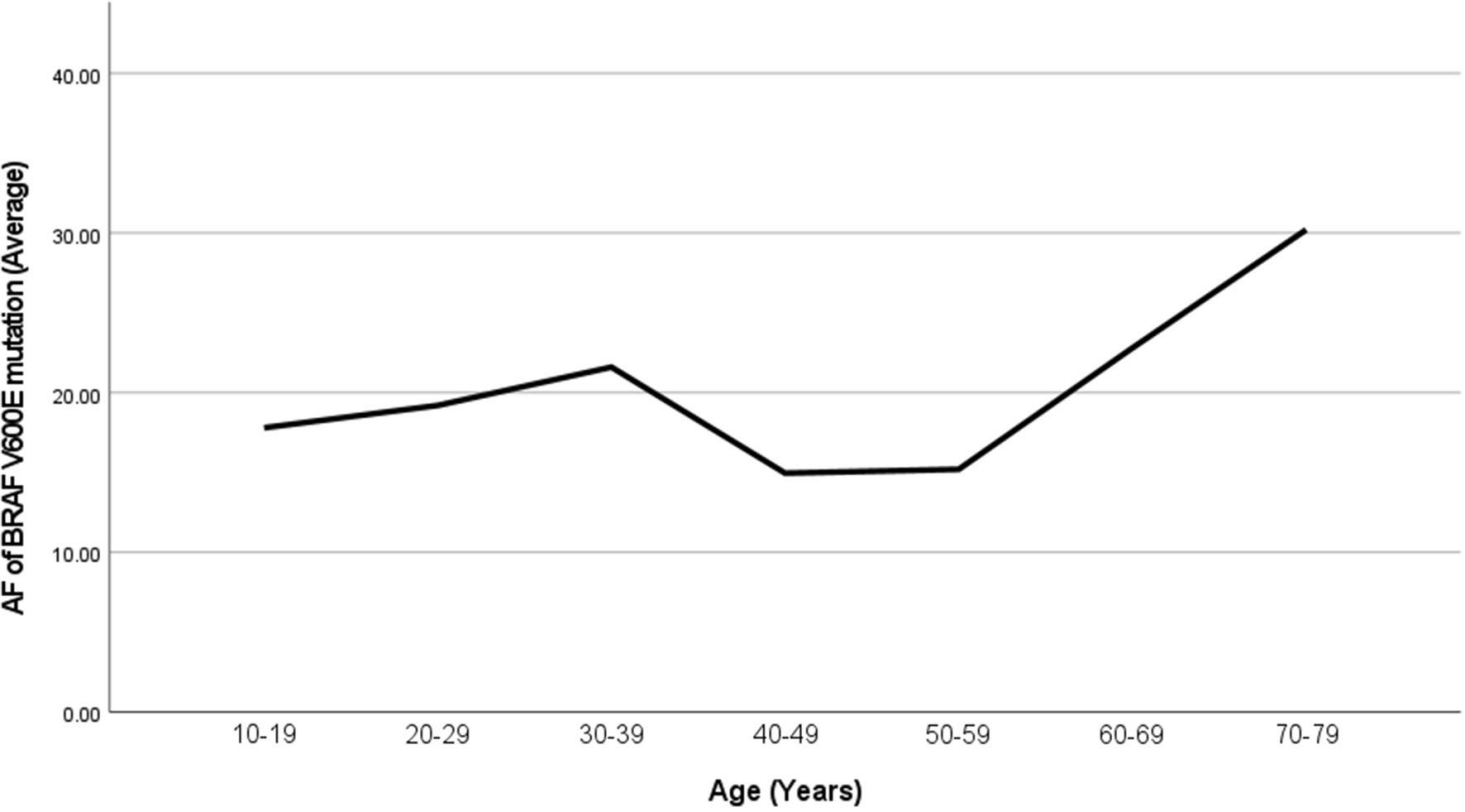
Line chart of *AF* of *BRAF V600E* mutation/Age (*n* = *75*)

The *BRAF V600E* mutation occurred in 75 cases (76.5%) of PTC patients, with a male-to-female ratio of 1:1.27. The average age was (44.89 ± 15.03) years, and the mean *BRAF V600E* mutation *AF* was (19.36 ± 11.27) %. For males, the average of *AF* was (19.09 ± 10.60%), and for females, it was (19.58 ± 11.89%). The difference in *BRAF V600E* mutation *AF* between males and females was not statistically significant (*p* = 0.87). The age distribution of average *BRAF* mutation frequency is shown in Table 2, and the trend is illustrated in Fig. 1, indicating a rise-fall-rise pattern with peaks at ages 30–39 years and 70–79 years groups. According to ATA and AJCC guidelines [13], age 55 is a critical point for AJCC staging. Therefore, the study population was divided into ≥ 55 years and < 55 years groups, showing no statistically significant difference in *BRAF* mutation *AF* between the two groups (*p* = 0.28).

**Table 2:** Average *BRAF V600E* Mutation *AF* Across Different Age Groups (*n* = *75*)

Age (years)	*BRAF V600E* mutation *AF* (%)
10–19	17.8
20–29	19.2
30–39	21.6
40–49	15.0
50–59	15.2
60–69	22.8
70–79	30.2

### Association between* AF *of *BRAF V600E* mutation and extrathyroidal extension

**Fig. 2:**
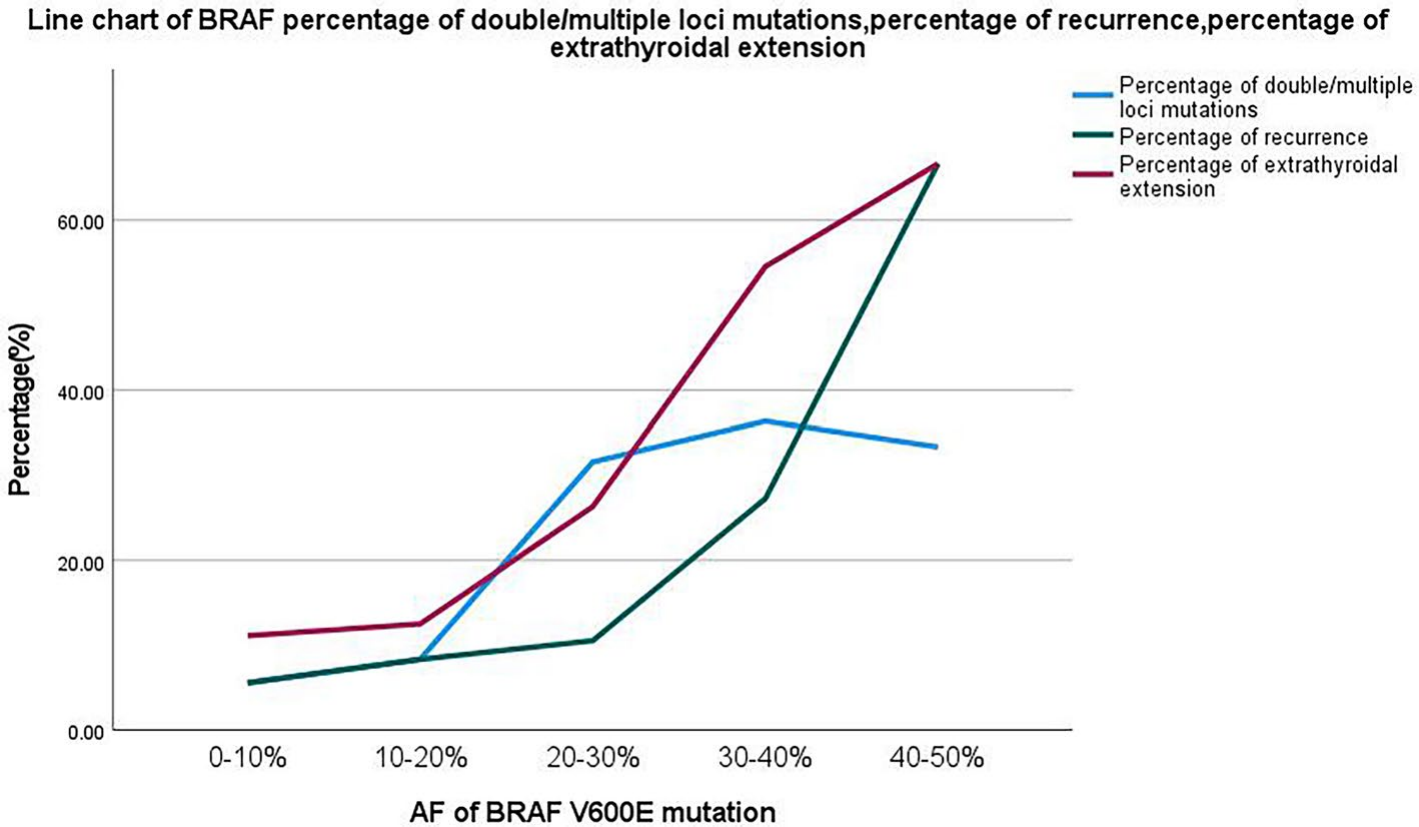
Line chart of *BRAF* percentage of double/multiple loci mutations, percentage of recurrence, percentage of extrathyroidal extension (*n* = *75*)

Among PTC patients with *BRAF V600E* mutations, 18 cases (24%) showed extrathyroidal extension breaking the thyroid capsule, as shown in Table 3. The line graph in Fig. 2 shows the relationship between the number of cases with thyroid cancer invasion and the *BRAF V600E* mutation *AF*. A significant association was found between extrathyroidal extension and the increased *AF* of *BRAF V600E* mutation. (*p* = *0.002*, OR = 1.100, OR (95%CI) = 1.037–1.166).

**Table 3:** Clinical Characteristics Across Different BRAF V600E Mutation AF (*n* = *75*)

*BRAF V600E* mutation AF	0–10%	10–20%	20–30%	30–40%	40–50%
Percentage of patients with thyroid cancer breaking through the thyroid capsule (%)	11.11	12.5	26.32	54.55	66.67
Percentage of recurrence (%)	5.56	8.33	10.53	27.27	66.67
Percentage of patients combined other mutations (%)	5.56	8.33	31.58	36.36	33.33

### Association between* AF *of *BRAF V600E* mutation and recurrence

**Fig. 3:**
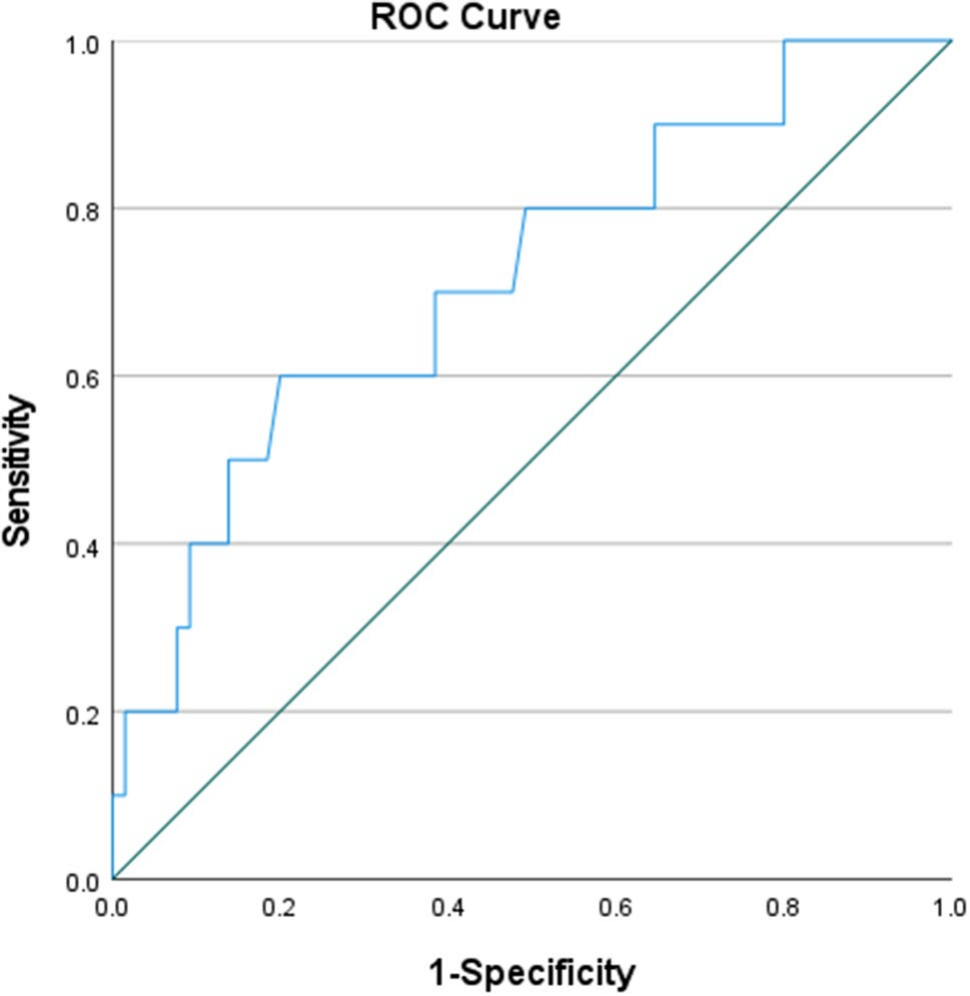
The ROC Curve for Predicting Recurrence Based on *BRAF V600E* Mutation *AF* (*n* = *75*)

Among PTC patients with *BRAF* mutations, 10 cases (13.3%) experienced recurrence, as shown in Table 3, with an average follow-up time of (1.86 ± 1.26) years. A significant association was found between *BRAF V600E* mutation *AF* and thyroid cancer recurrence (*p* = *0.023*, OR = 10.080, OR (95%CI) = 1.010–1.154). Figure 2 shows the line graph depicting the recurrence rate/*BRAF V600E* mutation *AF*. The receiver operating characteristic (ROC) curve was created using sensitivity on the y-axis and (1-specificity) on the x-axis. The area under the curve (AUC) was 0.717 (95% CI = 0.541–0.893), as shown in Fig. 3. The point with the highest Youden index was at a *BRAF V600E* mutation *AF* of 28.2% (sensitivity = 60.0%, specificity = 80.0%, Youden index = 0.40, accuracy=77.3%), as shown in Table 4. Table 5 shows that the risk of thyroid cancer recurrence is six times higher with a *BRAF V600E* mutation *AF* > 28.2% compared to *AF* ≤ 28.2%.

**Table 4:** Sensitivity and specificity of *BRAF V600E* mutation *AF* for predicting recurrence at various cutoff values (*n* = *75*)

*BRAF V600E* mutation *AF* (%)	Sensitivity	Specificity	Youden index
0.2	1	0	1
1.45	1	0.015	1.015
1.85	1	0.031	1.031
2.2	1	0.062	1.062
3	1	0.092	1.092
3.65	1	0.108	1.108
4.25	1	0.123	1.123
5.45	1	0.138	1.138
6.3	1	0.154	1.154
6.65	1	0.169	1.169
6.85	1	0.185	1.185
7.05	1	0.2	1.2
7.45	0.9	0.2	1.1
7.95	0.9	0.215	1.115
8.25	0.9	0.231	1.131
8.35	0.9	0.246	1.146
9.3	0.9	0.262	1.162
10.8	0.9	0.277	1.177
11.45	0.9	0.292	1.192
11.55	0.9	0.308	1.208
11.7	0.9	0.323	1.223
11.9	0.9	0.338	1.238
12.05	0.9	0.354	1.254
12.55	0.8	0.354	1.154
13.45	0.8	0.369	1.169
14.15	0.8	0.385	1.185
14.85	0.8	0.4	1.2
15.4	0.8	0.415	1.215
16.25	0.8	0.431	1.231
17.1	0.8	0.446	1.246
17.5	0.8	0.477	1.277
18.05	0.8	0.492	1.292
18.45	0.8	0.508	1.308
18.65	0.7	0.523	1.223
18.85	0.7	0.538	1.238
19.3	0.7	0.554	1.254
19.8	0.7	0.585	1.285
20.3	0.7	0.6	1.3
20.9	0.7	0.615	1.315
22	0.6	0.615	1.215
22.85	0.6	0.631	1.231
23.05	0.6	0.646	1.246
23.55	0.6	0.662	1.262
24.1	0.6	0.677	1.277
24.5	0.6	0.692	1.292
24.9	0.6	0.708	1.308
25.15	0.6	0.723	1.323
25.4	0.6	0.738	1.338
26.25	0.6	0.754	1.354
27.1	0.6	0.769	1.369
27.7	0.6	0.785	1.385
28.2	0.6	0.8	1.4
28.35	0.5	0.815	1.315
28.6	0.5	0.831	1.331
28.95	0.5	0.846	1.346
29.7	0.5	0.862	1.362
30.5	0.4	0.862	1.262
31	0.4	0.877	1.277
32.25	0.4	0.908	1.308
33.65	0.3	0.908	1.208
34.55	0.3	0.923	1.223
35.25	0.2	0.923	1.123
35.65	0.2	0.938	1.138
36.6	0.2	0.954	1.154
38.3	0.2	0.969	1.169
40.25	0.2	0.985	1.185
41.8	0.1	0.985	1.085
44.05	0.1	1	1.1
46.8	0	1	1

**Table 5:** Binary Logistic Regression Analysis of *BRAF V600E* Mutation *AF* > 28.2% and recurrence risk (*n* = *75*)

Relevance factor	*B*	*SE*	Wald	Freedom	*p *value	*OR (95%CI)*
*BRAF V600E* mutation *AF* > 28.2% or not	1.792	0.716	6.260	1	0.012	6.000(1.474 ~ 24.418)

### Association between* AF *of *BRAF V600E* mutation and combination with double/multiple loci mutations

Among PTC patients with *BRAF* mutations, 14 cases (18.67%) exhibited double/multiple loci mutations. These included 11 cases with *BRAF* + *TERT* mutations, 1 case with *BRAF* + *KRAS* mutation, 1 case with *BRAF* + *TERT* + *PIK3CA* mutations, and 1 case with *BRAF* + *TERT* + *AKT1* mutations. The likelihood of double/multiple mutations significantly increased with the elevated *BRAF* gene mutation frequency (*p* = *0.006*, OR = 1.088, OR (95%CI) = 1.024–1.156), as shown in Fig. 2, which depicts the relationship with *BRAF V600E* mutation AF. Compared to the group with a single BRAF mutation, the group with combined double/multiple *BRAF* mutations had a higher recurrence rate (*p* = 0.019, OR = 10.687, OR (95%CI) = 2.468–46.282), and a higher incidence of thyroid cancer breaking through the thyroid capsule (*p* < 0.001, OR = 16.562, OR (95%CI) = 4.178–65.662), as shown in Table 6.

**Table 6:** *BRAF* monoallelic mutation group Versus *BRAF* combined with other mutations group (*n* = *75*)

Relevance factor	*BRAF* monoallelic mutation group (*n* = 61)	*BRAF* combined with other mutations group (*n* = 14)	*p *value	OR	OR (95%CI)
Average *BRAF V600E* mutation *AF* (%)	(17.54 ± 10.75) %	(27.29 ± 10.31) %	0.006	1.088	1.040–1.156
Percentage of recurrence Patients (%)	6.6% (4/61)	42.9% (6/14)	0.002	10.687	2.468–46.282
Percentage of patients with tumor penetrating the thyroid capsule (%)	13.1% (8/61)	71.4% (10/14)	< 0.001	16.562	4.178–65.662

### Association between* AF *of *TERT* mutation and clinical statistics

*TERT* promoter mutations were present in 17 cases (17.3%) of PTC patients, with a gender ratio of 1:1.13 and an average age of (61.41 ± 10.86) years. The average *TERT* mutation frequency was (46.71 ± 11.09) %, and all mutations were *C228T* missense mutation. As shown in Table 7, 1 patient had a single-point mutation, 13 patients had double-point mutations, 2 patients had triple-point mutations, and 1 patient had a *TERT* gene mutation combined with ETV6-NTRK3 gene fusion. 7 patients (41.18%) experienced recurrence with a mean follow-up of (2.65 ± 5.17) years. Patients who experienced recurrence all had *TERT* mutation frequencies greater than 45%. There were no statistically significant differences in *TERT* mutation *AF* between different genders and age groups. Increasing *BRAF V600E* mutation *AF* leading to a significant increase in *TERT* mutations (*p* = *0.002*, OR = 1.116, OR (95%CI) = 1.042–1.197). Patients with *TERT* mutations have a significantly higher recurrence rate (*p* < *0.001, *OR = 12.429, OR (95%CI) = 2.805–55.064) and an increased risk of tumor breakthrough of the capsule (*p* < *0.001*, OR = 22.500, OR (95%CI) = 5.078–99.696), as shown in Table 8. There were no clear statistically significant differences in gender, age, thyroid capsule penetration, and recurrence among different *AF* of *TERT* mutation.

**Table 7:** *TERT* mutation types (*n* = *17*)

Groups (number)	*TERT* mutation types	Number (%)
TERT Monoallelic Mutation (*n* = 1)	*TERT* Mutation	1 (5.9%)
TERT Double Mutation Group (*n* = 13)	*TERT* + *BRAF* Mutation	12 (70.6%)
	*TERT* + *TP53* Mutation	1 (5.9%)
TERT Triple TERT Mutations (*n* = 2)	*TERT* + *NRAS* + *PIK3CA* Mutation	1 (5.9%)
	*TERT* + *BRAF* + *AKT1* Mutation	1 (5.9%)
TERT Combined with Gene Fusion (*n* = 1)	*TERT* Mutation + *ETV6-NTRK3* Fusion	1 (5.9%)

**Table 8:** BRAF monoallelic mutation group Versus *BRAF* + *TERT* mutations group (*n* = *75*)

Relevance factor	*BRAF* monoallelic mutation group (*n* = *61*)	*BRAF* + *TERT* mutations group (*n* = *13*)	*p* value	OR	OR (95%CI)
Average *BRAF V600E* Mutation *AF* (%)	(17.54 ± 10.75) %	(29.23 ± 7.61) %	0.002	1.116	1.042–1.197
Percentage of Recurrence Patients (%)	6.6% (4/61)	46.2% (6/13)	< 0.001	12.429	2.805–55.064
Percentage of Patients with Tumor Penetrating the Thyroid Capsule (%)	13.1% (8/61)	76.9% (10/13)	< 0.001	22.500	5.078–99.696

### Gene fusion

11 cases (11.22%) were detected with gene fusions, with a sex ratio of 1:1.75 and a mean age of (38.09 ± 16.15) years. The gene fusions included 7 cases of *CCDC6-RET* fusion, 2 cases of *NCOA4-RET* fusion, and 2 cases of *ETV6-NTRK3* fusion, as shown in Table 9. The *CCDC6-RET* fusion was found on chromosome 10, involving exon E1:E12 fusion. The *NCOA4-RET* fusion was found on chromosome 10, involving exon E8:E12 fusion. The *ETV6-NTRK3* fusion was found on chromosomes 12 and 15, involving exon E4:E14 fusion. The remaining 10 cases did not exhibit any combined gene mutations. Out of the 11 cases of gene fusion patients with papillary thyroid carcinoma, only one case showed a combination of *ETV6-NTRK3* fusion and *TERT* mutation. Among the gene fusion patients, two cases of *NCOA4-RET* fusion were of the Diffuse Sclerosing Variant of Papillary Thyroid Carcinoma (DSVPTC). Additionally, one patient with *ETV6-NTRK3* gene fusion experienced recurrence.

**Table 9:** Clinical characteristics of patients with gene fusion (*n* = *11*)

Gender	Age (years)	Gene Fusion	Pathology	Number of lymph node metastasis	Stage	Prognosis	Follow-up duration (Months)
Female	33	*CCDC6-RET*	PTC	12	pT1N1bM0	Survival, without Recurrence or Metastasis	3
Female	59	*NCOA4-RET*	DSVPTC	30	pT2N1bM0	Survival, without Recurrence or Metastasis	3
Male	28	*NCOA4-RET*	DSVPTC	16	pT4N1bM0	Survival, without Recurrence or Metastasis	10
Female	23	*CCDC6-RET*	PTC	29	pT2N1bM0	Survival, without Recurrence or Metastasis	15
Male	19	*CCDC6-RET*	PTC	13	pT2N1bM0	Survival, without Recurrence or Metastasis	17
Male	51	*CCDC6-RET*	PTC	0	pT2N1aM0	Survival, without Recurrence or Metastasis	3
Female	41	*CCDC6-RET*	PTC	17	pT2N1bM0	Survival, without Recurrence or Metastasis	17
Female	26	*CCDC6-RET*	PTC	16	pT1N1bM0	Survival, without Recurrence or Metastasis	16
Male	25	*CCDC6-RET*	PTC	20	pT4N1bM1	Survival, without Recurrence or Metastasis	40
Female	46	*ETV6-NTRK3*	PTC	5	pTxN1bM0	Survival, Recurrence	6
Female	68	*ETV6-NTRK3*	PTC	5	pT1N1bM0	Survival, without Recurrence or Metastasis	6

### Figures, tables and schemes

## Discussion

The incidence of PTC is higher in females than in males, with a ratio of 2–3 to 1. However, in patients with intermediate-to-high recurrence risk PTC, females are only 1.36 times than males, suggesting that more males develop intermediate-to-high risk PTC. Some studies suggested that male is considered one of the risk factors for lymph metastasis in thyroid cancer [14]. We observed no significant differences in the allele frequencies of *BRAF V600E* and *TERT* mutations between males and females, which indicates that the severity of thyroid cancer in males may be associated with other factors. The occurrence rates of *TERT* mutations in thyroid cancer do not differ significantly among Asians, Europeans, and Americans [9, 10]. However, the occurrence rate of *BRAF V600E* mutations varies significantly across different races. The incidence of *BRAF V600E* mutation varies among different populations. In American patients with PTC, the incidence is approximately 50.8% [3]. In Korean patients, the incidence is around 72% [4], while in Japanese patients, it is about 38% [5]. In Italian patients, the incidence is around 38.1% [7], and in Saudi Arabian patients, it is about 59.5% [6]. In Chinese PTC patients, the incidence is approximately 72.4% [11], which is similar to the rate observed in our retrospective analysis (76.5%). Although *BRAF V600E* is considered a major driver in Chinese PTC populations, *BRAF V600E AF* in Chinese PTC patients is not significantly higher than in other regions and is even lower in our study. The reported *BRAF V600E AF* in PTC in other regions is approximately 26% in Canada [12] and around 27% in Italy [15]. While our study found that the *AF* of *BRAF V600E* in Chinese patients was approximately 19.36%, demonstrating inconsistency between its *AF* and incidence characteristics.

Gene mutations and fusions often do not occur simultaneously, exhibiting mutual exclusivity. Only 1 patient was found to have both *TERT* mutation and *ETV6-NTRK3* fusion, but this patient's condition was not severe. Among the patients analyzed, the most common gene fusion was *CCDC6-RET* fusion (63.6%), followed by *NCOA4-RET* fusion (18.2%) and *ETV6-NTRK3* fusion (18.2%), which may be related to a history of neck exposure [16, 17].

The *AF* of *BRAF V600E* mutation is suspected to be associated with metastasis in PTC, leading to higher tumor staging and poor outcome [5, 11, 12]. However, some studies have contradicted this claim, stating that the *AF* of *BRAF V600E* mutations does not significantly impact the prognosis and invasiveness of PTC [15, 18]. These discrepancies may be related to the diversity of populations in different countries or variations in the risk stratification of patients. Tumors form due to genomic instability in somatic cells, which can lead to the emergence of aggressive clones that can survive and outcompete other cells in the microenvironment. With the competition among cells, various genomic compositions (Allele Frequency) have emerged. The overall density of somatic mutations is relatively low, and this is considered the biological basis for the indolent clinical behavior observed PTC [18–21]. The efficacy of *AF* varies in different literature, which may be due to its unclear efficacy in low-risk thyroid cancer patients, while its effect becomes more distinct in intermediate-to-high-risk patients, clearly demonstrating its association with the disease. Therefore, we focused on intermediate-to-high-risk population to avoid interference from the large low-risk PTC population. In addition, genetic testing is a costly procedure, and although we found that *BRAF V600E AF* can predict patient prognosis, it is apparently unnecessary for low-risk PTC patients, since next generation sequencing techniques are mainly applicable to populations with intermediate-to-high risk recurrence patients. However, as this is a single-center, single-region, single-ethnicity study, it has certain limitations. Future research could include multiple populations, regions, and larger sample sizes to further investigate the overall impact of *BRAF* and *TERT* gene frequencies on disease. In addition, 94.1% of *TERT* mutation carriers had other types of genetic alterations, which may be due to the small sample size of *TERT* mutations. The lack of significant association between *TERT* promoter mutation *AF* and disease prognosis may be influenced by other mutation types, which requires further investigation.

We analyzed the impact of *BRAF V600E* mutation *AF* on tumor invasion, co-occurring mutations, and recurrence in patients with PTC in intermediate and high-risk recurrence categories. For each 1% increase in *BRAF V600E* mutation frequency, the risk of tumor invasion, recurrence, and co-occurring gene mutations increases by 10.0%, 8.0%, and 8.8%, respectively. The combination of BRAF mutation with other gene mutations significantly increases the risk of thyroid cancer invading the thyroid membrane and recurrence, with *TERT* mutations being the most common co-occurring mutation. When the frequency of the *BRAF V600E* mutation is greater than 28.2%, the risk of thyroid cancer recurrence increases sixfold compared to when the frequency is 28.2% or lower. Using *AF* of *BRAF V600E* mutation > 28.2% to predict the recurrence of intermediate and high risk PTC has a sensitivity of 60% and a specificity of 80%, making it a potential new indicator for predicting the risk of thyroid cancer recurrence.

The study indicates that BRAF mutations are the primary genetic mutation events in PTC, followed by *TERT* mutations, while other types occur less frequently. *TERT* mutations are associated with older age (average 61.41 years) and a higher *AF* (average 46.71%). Research indicates that *TERT* mutation is infrequent in children and teenagers [22], while combined with BRAF mutation, they contribute to increased malignancy in thyroid cancer [23, 24]. Our study suggested that *TERT* mutations are often accompanied by other mutations. The higher malignancy of *TERT* mutations in PTC may be due to the co-occurrence of other mutations [25]. The co-occurrence of *BRAF* mutations with *TERT* mutations is the most common scenario, and there is a positive correlation between the *AF* of *BRAF V600E* and the risk of co-occurring *TERT* mutations. According to extensive data reviewed by scholars, there appears to be no significant correlation between *BRAF* mutation and distant metastasis in PTC patients, while *TERT* mutation has been associated with distant metastasis. But our study identified a potential association between a high allele frequency of *BRAF V600E* and *TERT* mutation, which is consistent with the findings of previous research, *BRAF* mutations may lead to abnormal overexpression of the *TERT* promoter [26]. However, among the 94 patients we reviewed, none showed evidence of distant metastasis. Further research is needed to explore the relationship between *BRAF*, *TERT* mutations and distant metastasis [27].

## Conclusions

Although the incidence of *BRAF V600E* mutation varies across different regions, the *AF* of *BRAF V600E* mutation is similar between Asian and Western patients. *AF* of *BRAF V600E* is positively correlated with invasiveness and the risk of recurrence. It may induce other gene mutations, such as *TERT* mutations, thereby enhancing the invasive capabilities of the tumor and leading to a poorer prognosis. The *AF* of *BRAF V600E* shows potential as a novel indicator for predicting tumor invasiveness and prognosis.

## Data Availability

The data that support the findings of this study are available from the corresponding author upon reasonable request.

## Abbreviation

AF: Allele frequency
